# Delivery Progress, Labor Interventions and Perinatal Outcome in Spontaneous Vaginal Delivery of Singleton Pregnancies between Nulliparous and Primiparous Women with One Previous Elective Cesarean Section: A Retrospective Comparative Study

**DOI:** 10.3390/life13102016

**Published:** 2023-10-05

**Authors:** Grigorios Karampas, Martin Witkowski, Dimitra Metallinou, Margareta Steinwall, Alkis Matsas, Theodoros Panoskaltsis, Panagiotis Christopoulos

**Affiliations:** 1 Department of Obstetrics and Gynecology, Skånes University Hospital, 21428 Malmö-Lund, Sweden; 2 Second Department of Obstetrics & Gynecology, Medical School, University of Athens “Aretaieion” Hospital, 11528 Athens, Greece; 3 Department of Obstetrics and Gynecology, Kristianstad/Ystad Community Hospitals, 27133 Ystad, Sweden; 4 Department of Midwifery, University of West Attica, 12243 Athens, Greece

**Keywords:** TOLAC, VBAC, singleton pregnancy, nulliparous, primipara, spontaneous labor, delivery progress, perinatal outcome

## Abstract

Trial of labor after cesarean (TOLAC) is an alternative to repeated cesarean for women with singleton pregnancy and one previous transverse lower segment cesarean section (LSCS), resulting in most cases being a successful vaginal birth after cesarean section (VBAC). The primary objective of this study was to examine if the progress and the duration of the active first stage and the second stage of labor in nulliparous women with singleton pregnancy, spontaneous start of labor and vaginal birth differ from primiparous women succeeding VBAC after one previous elective LSCS in a country with a low cesarean section and high VBAC rate. Secondary objectives were to compare labor interventions and maternal–neonatal outcomes between the two groups. Methods: This is a retrospective comparative study. Data were collected in a four-year period at the departments of Obstetrics and Gynecology at Kristianstad and Ystad hospitals in Sweden. Out of 14,925 deliveries, 106 primipara women with one previous elective LSCS and a spontaneous labor onset in the subsequent singleton pregnancy were identified. Of these women, 94 (88.7%) delivered vaginally and were included in the study (VBAC group). The comparison group included 212 randomly selected nulliparous women that had a normal singleton pregnancy, spontaneous labor onset and delivered vaginally. Results: The rate of cervical dilation during the active first stage of labor as well as the duration of the second stage did not differ between the two groups. When adjusting for cervical dilation at admission, there was no significant difference between the two groups regarding the duration of the active phase of the first stage of labor. No significant differences were found in maternal–neonatal outcomes between the two groups except for higher birth weight in the VBAC group. The use of epidural analgesia was associated with slower dilation rhythm over the duration of the active phase and second stage of labor, need for labor augmentation, postpartum bleeding and need for transfusion at higher rates, irrespective of parity when epidural was used. Conclusions: Our study provides evidence that in women with one previous elective LSCS undergoing TOLAC in the subsequent pregnancy resulting in vaginal birth, the progress and duration of labor are not different from those in nulliparous women when labor is spontaneous and the it is a singleton pregnancy. The use of epidural was associated with prolonged labor, need for labor augmentation and higher postpartum bleeding, irrespective of parity. This information may be useful in patient counseling and labor management in TOLAC.

## 1. Introduction

Cesarean section (CS), when indicated, is a lifesaving procedure both for the mother and the fetus [1,2]. However, it results in prolonged hospital stay and increases maternal and neonatal morbidity in subsequent deliveries [3,4]. Though low, the frequency of severe complications in subsequent pregnancies after the first CS, such as placenta previa increta/percreta, placenta abruption, uterine rupture and intrauterine fetal death (IUFD), is increased compared to vaginal delivery [5,6,7].

The ideal CS rate is still debatable, but according to the World Health Organization (WHO), a rate of 10–15% is considered optimal [8]. Unfortunately, since the 70’s, the CS rate in many developed countries, such as the United States of America, has increased significantly from 5% to approximately 32% [9]. By contrast, the CS rate in the Scandinavian countries has increased more moderately [10]. In Sweden, the CS rate increased from 11% in 1990 to 17% in 2013 and has remained close to the WHO recommendation level since then [10,11].

In an effort to reduce CS rates, many countries introduced trial of labor after cesarean delivery (TOLAC) as a choice for most women with one previous transverse lower (uterine) segment cesarean section (LSCS), frequently resulting in a successful vaginal birth after cesarean section (VBAC) [12]. Nevertheless, TOLAC and VBAC rates have progressively decreased in many countries as the optimal mode of delivery for women who have had one previous LSCS remains controversial [13,14,15,16,17]. Sweden belongs, though, to the group of countries with a high overall TOLAC rate of approximately 69% resulting in an overall VBAC rate of 49.6% [11,17]. Thus, approximately half of the women in Sweden with one previous LSCS preform a VBAC, irrespective of prior CS indication [17].

Many studies have investigated predictive factors for a successful TOLAC as well as the total success rate [18,19,20]. Nonetheless, there is limited data on labor progress in specific subgroups of women undergoing a TOLAC, such as primiparous women with one prior elective CS and spontaneous labor onset, as in most previous studies, women that underwent a TOLAC were treated as one general group without any further subgroup analysis based on specific maternal characteristics [21,22,23]. As the decision for a TOLAC should be based on individualized risk assessment, success rate and informed consent, it is important to understand labor patterns in subsequent deliveries in specific subgroups of candidates [24,25]. Understanding labor patterns among different subgroups of women with one LSCS trying a TOLAC will help in the counseling and the clinical management of labor in these women.

The primary aim of the present study was to compare delivery progress in singleton pregnancies between nulliparous women that delivered vaginally after a spontaneous labor start and a specific VBAC subgroup consisting of primiparous women with only one previous elective LSCS without previous labor nor induction that should also have a spontaneous start of labor in the second pregnancy. Secondary outcomes were labor interventions and perinatal outcome.

## 2. Materials and Methods

This is a retrospective comparative study, which was conducted over the period of January 2013 to May 2017 at the department of Obstetrics and Gynecology, Kristianstad and Ystad general community hospitals in Sweden.

Inclusion criteria for the VBAC group were primiparous women with one previous elective LSCS at >35 weeks of gestation without any induction trial or spontaneous start of labor in their first pregnancy with a singleton full-term (>37 weeks) subsequent pregnancy and spontaneous labor onset (TOLAC) resulting in VBAC. Inclusion criteria for the comparison group were nulliparous women with a singleton full-term (>37 weeks) pregnancy and spontaneous labor onset resulting in vaginal delivery. Exclusion criteria for both groups were ongoing severe preeclampsia, fetal growth restriction (FGR), insulin-dependent diabetes mellitus (IDDM), recurrent CS, previous emergency CS during labor or previous induced labor. Data were extracted from antenatal and hospital charts. Data loss was minimal or absent since all visits were covered via the public health care system and charts are registered precisely.

**Figure 1:**
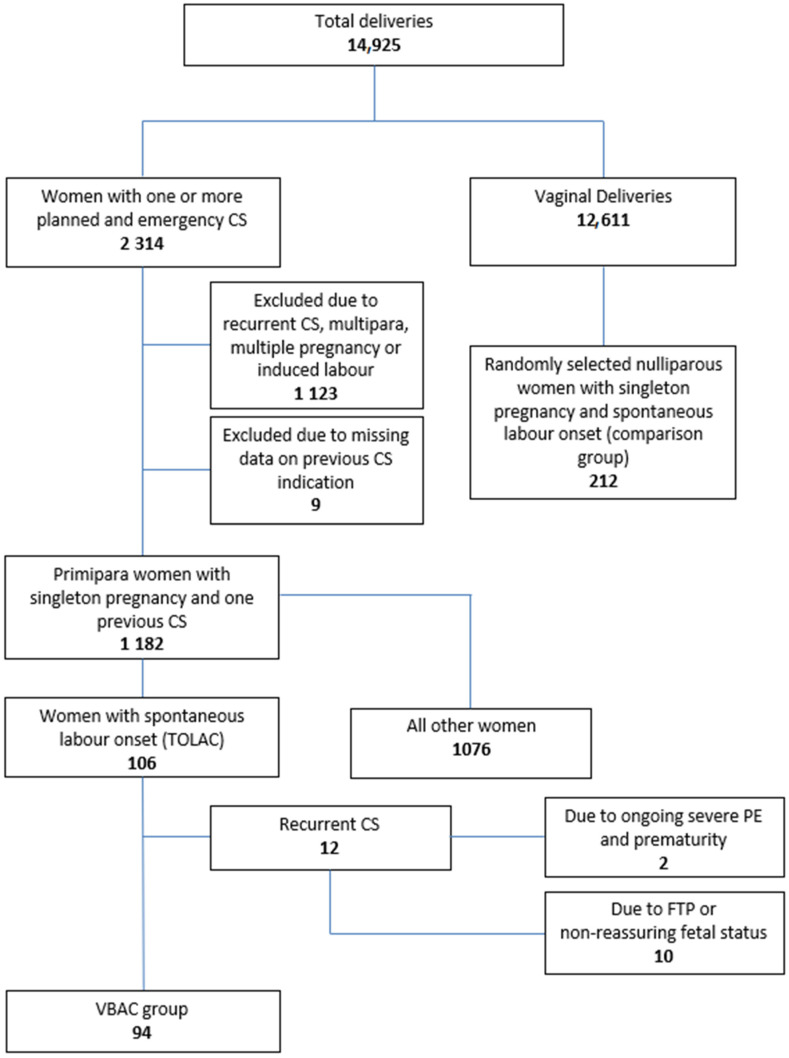
Flowchart. CS: cesarean section; TOLAC: trial of labor after cesarean; VBAC: vaginal birth after cesarean; FTP: failure to progress.

In total, 14,925 deliveries and an overall CS rate of 15.5% were recorded over the study period (Figure 1).

Of all deliveries, 2314 women had one or more previous elective/emergency CS from which 1182 had one previous CS. From the latter, 106 were primipara with one previous elective LSCS and a spontaneous labor onset in the subsequent singleton normal pregnancy (TOLAC). Of these women, 94 (88.7%) had a successful TOLAC resulting in VBAC and were included in the study (VBAC group), while the remaining 12 had an unsuccessful TOLAC resulting in repeat cesarean delivery (Figure 1). From the VBAC group, 69 women had CS due to prior breech presentation; 13 women, due to past-pregnancy-related complications (such as pre-eclampsia, placenta previa and twin pregnancy); 6 women, due to maternal request; and 6 women, for other indications (such as suspected fetal macrosomia).

For every woman in the VBAC group two nulliparous women with a normal full-term singleton pregnancy, who had a spontaneous labor onset and delivered vaginally, were randomly selected and included in the comparison group (*n* = 212).Women in the comparison group were randomly selected by including the first two women that fulfilled the inclusion criteria from each of the two hospitals (4 women in total) each month over the study period.

We defined the start of the active phase of the first stage of labor when two out of the following three criteria were met [26,27,28,29]:

- At least 4 cm dilated cervix;
- Minimum 3 painful contractions per 10 min;
- Ruptured membranes.

The second stage of labor was defined as the period from full dilation to delivery, and the third stage as the period following the delivery of the newborn until the delivery of the placenta [27,28].

### Statistical Analyses

The statistical software IBM SPSS statistics version 23 (IBM Corporation, Somers, NY 10589, USA) was used for data analysis. One-sample Kolmogorov–Smirnov test was used to examine the normality of the distribution of quantitative parameters in the two study groups. For quantitative parameters with normal distribution, Student’s *t*-test was used for comparison between the groups, while Mann–Whitney U-test was used for variables that were not normally distributed. Comparisons of qualitative parameters were performed using Pearson’s chi-square test (X^2^). For sub-group analysis, the comparison of quantitative characteristics among the groups was performed with the use of either one-way ANOVA or Kruskal–Wallis test, depending on the normality of their distribution. A univariate analysis of covariance (ANCOVA) was performed by setting the duration of the active phase of the first stage of labor as the dependent variable, adjusting the two groups for cervix dilation at admission in order to control if there was a difference in labor progress. A probability level of less than or equal to 0.05 was considered significant.

Ethical approval: The present study was conducted in accordance with the Declaration of Helsinki and approved by the Regional board of research and ethics, Lund University (EPN DNR 2017/556).

## 3. Results

Maternal characteristics are presented in Table 1.

The two groups differed significantly in maternal age, but were comparable on other factors. Neonates in the case group were significantly heavier despite the congruence on week of labor and maternal weight (Table 1). Women in the VBAC group received significantly more oxytocin postpartum than nulliparous women, while no significant difference was seen in total amount of bleeding nor in the need for blood transfusion (Table 2).

Overall, no significant difference was observed between the groups in the use of forceps/vacuum extractor, epidural analgesia, the use of oxytocin in the active phase of the first stage of labor as well as in the second stage, nor in the use of other uterotonic agents postpartum.

Regarding labor progress, a significant difference in the duration of the active phase of the first stage of the labor between the two groups was detected, but this difference was expected as women with one previous CS were admitted to the labor ward presenting a lower cervix dilation status (mean 5 cm) than nulliparous (mean 6 cm). Consequently, we performed a univariate analysis of covariance (ANCOVA) by setting the duration of the active phase of the first stage of labor as the dependent variable, adjusting the two groups for cervix dilation at admission in order to control if there was a difference in labor progress. The final analysis revealed no significant difference between the two groups regarding the active phase and the second stage of labor. Additionally, a comparison of cervix dilation rhythm confirmed that there was no difference between the two groups.

Subgroup analysis among nulliparous women with (*n* = 160) or without (*n* = 52) and primiparous women with (*n* = 67) or without (*n* = 27) the use of epidural during labor (Table 3 and Table 4) showed that, except for maternal age, there was no other discrepancy among the four subgroups regarding maternal characteristics (Table 3).

On the contrary, there was a significant difference among the four groups on cervix dilation at admission, the duration of the active phase of the first labor stage, dilation rhythm, the duration of the second labor stage, the birthweight of the newborn and the amount of postpartum bleeding (Table 4).

More precisely, the duration of the active phase and the second stage of labor were longer, while dilation rhythm was slower in women that had epidural during labor, irrespective of parity. Moreover, the birthweight of the newborn was higher in primiparous women that had epidural when compared to nulliparous women without epidural, whereas postpartum bleeding was higher in nulliparous women with epidural in comparison to nulliparous without epidural. Finally, the use of oxytocin during the active phase and the second stage of labor were more frequent in women that had epidural during labor than in those without, irrespective of parity, while the need for transfusion was more frequent in nulliparous women with epidural than in nulliparous without epidural during labor.

## 4. Discussion

It is well documented that spontaneous labor onset and advanced cervical dilatation at admission to the labor ward are among the most significant factors associated with successful TOLAC resulting in VBAC in women with one previous LSCS [29]. Nevertheless, studies specifically comparing labor progress and intrapartum management between nulliparous women with a spontaneous vaginal delivery to primiparous women with one elective LSCS and spontaneous labor onset resulting in VBAC in the subsequent pregnancy are limited in the literature [22,30,31,32]. As the rate of elective CS in nulliparous women rise in many countries [8,10] it is essential for healthcare professionals to be able to provide evidence-based information to women concerning labor patterns and interventions when TOLAC is desirable and not contraindicated.

In the present study, we demonstrated that the success rate of TOLAC in full-term singleton pregnancies is quite high (88.7%) in women with one prior elective LSCS when labor is spontaneous. The success rate resembles that of the vaginal delivery rate of full term singleton pregnancies in nulliparous women with spontaneous labor onset in Sweden (81.4%) [33]. This finding is in accordance to our results as there was no significant difference in the delivery progress between the two groups. In detail, dilation rhythm in the active phase of the first labor stage, the duration of the second labor stage, the use of oxytocin in the first and second labor stage, epidural and need for instrumental delivery appeared similar. Thus, labor patterns are similar between the two groups, with an average dilation speed of more than one centimeter per hour, a finding which is also consistent with the literature of mean dilation rhythm in nulliparous women of no less than 1.2 cm/h [27,28,31,33]. Additionally, the average duration of the second labor stage in both groups lasted less than three hours, finding, which is in line with previous studies [27,28,29,32].

The observation that women in the case group were admitted to the labor ward at a lower cervix dilation status was expected since women with previous LSCS undergoing a TOLAC are considered to represent a higher obstetric risk population in comparison to nulliparous women [34]. On the other hand, the more frequent use of oxytocin in the third stage of labor in women with VBAC is expected as previous studies have demonstrated its protective role for postpartum hemorrhage (PPH) [35].

Additionally, no significant difference was detected regarding maternal–neonatal outcomes and labor interventions of interest; such as the need for instrumental delivery; postpartum bleeding; the use of uterotonics and transfusion; as well as severe adverse perinatal outcomes, such as stillbirth beyond 39 weeks, uterine rupture, maternal/neonatal mortality and neonatal brain injury; even if neonates in the VBAC group were significantly heavier than those in the comparison group. The later finding is also consistent with previous studies as neonates of multiparous women are heavier than these of nulliparous women [36]. Finally, women in the case group were older, which is a reasonable finding taking into consideration the completion of a previous full term pregnancy and maternal leave of absence until the next pregnancy.

As the use of epidural and its effects on labor progress and perinatal outcome are still debatable in women undergoing TOLAC [37], a subgroup analysis took place among women in the two groups in respect to the use or not of epidural during labor (Table 3 and Table 4). In all cases, women that received epidural had a longer active phase of the first labor stage, lower cervix dilation rhythm and longer second labor stage when compared to women that did not use epidural during labor, irrespective of parity status. Moreover, the need for labor augmentation in both the active phase and the second stage of labor was more frequent in the two subgroups of women that had epidural compared to those without. Our findings align well with previous studies [38,39], in which the use of epidural was associated with a prolonged second labor stage. However, it is worth mentioning that no significant difference was observed regarding labor patterns between the subgroups of nulliparous and primiparous women that had epidural during labor.

As this is a retrospective comparative study there are specific strengths and limitations. An important strength of our study is the high quality of our data and, consequently, our results, as missing data are limited due to the high quality of documentation. Moreover, the possibility of co-founding factors is restricted due to the fact that Sweden (a) is one of the few countries worldwide with a very low overall CS rate in combination with excellent perinatal outcome and high VBAC rate, and (b) has centralized medicaldata documentation system, (c) zero private-section deliveries, (d) common and frequent continuing medical education for obstetric staff, and (e) centralized guidelines on pregnancy follow-up, labor monitoring, and induction/augmentation methods and interventions that ensure a high level of homogeneity regarding the provided healthcare among different hospitals and medical staff. Another advantage is that due to the retrospective nature of our study, it has high reliability with the everyday clinical practice in middle-class community hospitals in Sweden, indicating that TOLAC and VBAC are feasible options not only for tertiary hospitals, but also for secondary ones under specific conditions regarding facilities and medical staff education. Moreover, as the study personal had no former information on the purpose of the study, no selection bias or extra care of women included in the study took place. Finally, by focusing on primiparous women with one prior elective CS, we present data that refer to a progressively higher percentage of women, with one prior elective CS seeking individualized information on a subsequent TOLAC.

On the contrary, specific limitations due to the retrospective design of this study are inevitable. Hence, as no prior data are available specifically comparing the selected subgroups of women used in our study, no prior sample size calculation could take place. Nonetheless, a post hoc sample size calculation, using the “Stratulator” free online software for the “Sample Size Calculator for Comparing Two Independent Means”, revealed that in order to detect a significant difference of 0.3 cm/hour between the two groups on cervix dilation rhythm, a total of 669 women in each group would be necessary. Thus, that difference would represent approximately a one hour discrepancy for the completion of the active phase of the first labor stage between the two groups, a finding which has limited significance in every day clinical practice. Likewise, a post hoc sample size calculation on the duration of the second labor stage would require 5258 women in each group in order to detect a difference of twenty minutes between the two groups. Based on those results, our study provides evidence that labor patterns are similar between the two groups of pregnant women.

Another important limitation of our study is the small number of women included in the case group in terms of adverse perinatal outcome and more specifically regarding uterus rupture. It is well known that the overall frequency of uterine rupture in TOLAC after one previous CS is approximately 0.5% [25] or even lower (0.15–0.4%) [39] when labor is spontaneous, and consequently, our study is underpowered to detect differences in uterine rupture between the two groups. The same is true for other more rare complications of TOLAC, such as the need for hysterectomy, antepartum stillbirth, hypoxic ischemic encephalopathy (HIE) and maternal/neonatal perinatal mortality.

Therefore, future larger retrospective or prospective comparative studies or, ideally, prospective randomized control trials (RCTs) comparing different options of labor management are of great interest in order to confirm our findings on delivery progress and to determine safety and perinatal outcomes in singleton pregnancies with spontaneous labor onset between nullipara and women with one prior elective LSCS undergoing TOLAC. As the rate of elective CS in nulliparous women is rising worldwide, and informed consent of women attempting TOLAC in the subsequent pregnancy must be based on evidence, our findings need confirmation by larger retrospective studies or prospective RCTs especially in terms of perinatal safety.

## 5. Conclusions

According to our findings, delivery progress and labor patterns in women with one previous elective LSCS performing TOLAC in the subsequent pregnancy are similar to those of nulliparous women when labor is spontaneous and it is a singleton pregnancy. The use of epidural was associated with prolonged labor, need for labor augmentation and higher postpartum bleeding, irrespective of parity.

## Figures and Tables

**Table 1:** Mean ± standard deviation (SD), median (interquartile range (IQR)) and frequencies of maternal characteristics in the two groups.

	Nullipara (*n* = 212)			VBAC (*n* = 94)			*p*-Value
	*n* (%)	Mean ± SD	Median (IQR)	*n* (%)	Mean ± SD	Median (IQR)	
**Age (years)**		28.1 ± 4.6	27.5 (5.8)		29.9 ± 4.7	29.1 (5.9)	0.001
**BMI 1st** **trimester (kg/m^2^)**		24.1 ± 4.1	23.1 (5.1)		25.1 ± 4.6	24.8 (5.9)	0.062
**BMI 3rd trimester (kg/m^2^)**		29.0 ± 4.3	28.0 (5.1)		29.8 ± 4.6	29.7 (5.5)	0.108
**Week of labor**		39.8 ± 1.2	39.8 (1.7)		40.0 ± 1.1	40.1 (1.5)	0.094
**Abortion or misscarricage**	71/212 (33.5)			94/94 (100)			<0.001
**Smoking**	13/203 (6.4)			7/90 (7.8)			0.667
**Alcohol**	1/201 (0.5)			4/85 (4.7)			0.013

**Table 2:** Mean ± standard deviation (SD), median (interquartile range (IQR)) and frequencies of labor characteristics in the two groups.

	Nullipara (*n* = 212)			VBAC (*n* = 94)			*p*-Value
	*n* (%)	Mean (95% CI)	Median (IQR)	*n* (%)	Mean (95% CI)	Median (IQR)	
**Cervix dilation at admission (cm)**		5.7 ± 2.1	5.0 (3.0)		4.8 ± 1.9	4.0 (2.0)	0.001
**Duration active phase (h)**		3.51 ± 3.2	2.62 (4.33)		4.81 ± 3.8	3.62 (4.61)	0.005 *
**Dilation rythm** **(cm/h)**		2.46 ± 3.55	1.53 (2.06)		2.1 ± 2.24	1.28 (1.49)	0.192
**Duration second stage (h)**		2.43 ± 1.8	1.95 (2.35)		2.33 ± 1.9	1.81 (2.48)	0.372
**Birthweight (gr)**		3501 ± 412	3465 (528)		3636 ± 465	3635 (649)	0.016
**Post-partum bleeding (mL)**		560 ± 413	425 (300)		549 ± 411	400 (306)	0.806
**Oxytocine active phase**	36/212 (17.0)			16/94 (17.0)			0.993
**Oxytocin 2nd stage of labor**	97/212 (45.8)			40/93 (43.0)			0.657
**Oxytocin 3rd stage of labor**	46/212 (21.7)			31/94 (33.0)			0.036
**Epidural**	52/212 (24.5)			27/94 (28.7)			0.439
**Instrumental delivery**	23/212 (10.8)			13/94 (13.8)			0.455
**Post-partum medicine**	23/212 (10.8)			14/94 (14.8)			0.317
**Transfusion**	18/212 (8.5)			7/94 (7.4)			0.758
**Uterine rupture**	0/212			0/94			NM
**Antepartum** **stillbirth beyond 39^+0^ weeks**	0/212			0/94			NM
**Intra/postpartum maternal/neonatal mortality**	0/212			0/94			NM
**HIE**	0/212			0/94			NM

Post-partum medicine (metylergometrine, additional oxytocine, prostaglandines and/or tranexamic acid). HIE: Hypoxic ischemic encephalopathy; NM: not meaningful. * When the two groups were adjusted for cervix dilation at admission (ANCOVA analysis), no significant difference in the duration of the active phase of the first labor stage was detected between the two groups.

**Table 3:** Mean ± standard deviation (SD), median (interquartile range (IQR)) and frequencies of maternal characteristics in women with and without epidural during labor in respect to parity.

	Nullipara						VBAC						*p*-Value
	No Epidural (*n* = 160)			Epidural (*n* = 52)			No Epidural (*n* = 67)			Epidural (*n* = 27)			
	*n* (%)	Mean ± SD	Median (IQR)	*n* (%)	Mean ± SD	Median (IQR)	*n* (%)	Mean ± SD	Median (IQR)	*n* (%)	Mean ± SD	Median (IQR)	
**Age (years)**		28.35 ± 4.69	27.66 (6)		27.5 ± 4.14	27.5 (7)		29.46 ± 4.52	28.91 (5)		30.98 ± 5.12	31.12 (7)	0.005 *
**BMI 1st** **trimester (kg/m^2^)**		23.9 ± 3.99	23.05 (5.05)		24.6 ± 4.5	23.97 (6.08)		25.1 ± 4.75	25.1 (6.05)		25.96 ± 4.23	24.45 (4.91)	0.222
**BMI 3rd trimester (kg/m^2^)**		28.65 ± 3.99	27.8 (5.22)		30.06 ± 4.99	29.4 (6.56)		29.78 ± 4.74	29.75 (6.02)		29.91 ± 4.21	28.84 (5.54)	0.123
**Week of labor**		39.76 ± 1.18	39.7 (1.72)		39.88 ± 1.13	39.84 (1.84)		40.08 ± 1.01	40.14 (1.42)		39.95 ± 1.29	40.14 (2.11)	0.33

* *p* = 0.048 between nullipara without and primipara with epidural (Bonferroni post hoc test). *p* = 0.024 between nullipara and primipara with epidural (Bonferroni post hoc test).

**Table 4:** Mean ± standard deviation (SD), median (interquartile range (IQR)) and frequencies of labor characteristics in women with and without epidural during labor in respect to parity.

	Nullipara						VBAC						*p*-Value
	No Epidural (*n* = 160)			Epidural (*n* = 52)			No Epidural (*n* = 67)			Epidural (*n* = 27)			
	*n* (%)	Mean ± SD	Median (IQR)	*n* (%)	Mean ± SD	Median (IQR)	*n* (%)	Mean ± SD	Median (IQR)	*n* (%)	Mean ± SD	Median (IQR)	
**Cervix dilation at admission (cm)**		6.0 ± 2.2	5.5 (3.0)		4.5 ± 0.9	4.0 (1.0)		4.9 ± 2.1	5.0 (2.0)		4.5 ± 1.4	4.0 (1.5)	<0.001 *
**Duration active phase (h)**		2.82 ± 2.81	1.87 (3.36)		5.77 ± 3.45	5.25 (5.52)		3.88 ± 3.22	3.25 (3.26)		7.08 ± 4.24	6.58 (6.52)	<0.001 **
**Dilation rythm** **(cm/h)**		2.85 ± 4.05	1.91 (2.49)		1.44 ± 1.22	1.01 (0.9)		2.46 ± 2.43	1.7 (1.85)		1.28 ± 1.46	0.8 (0.74)	<0.001 ***
**Duration second stage (h)**		2.24 ± 1.74	1.77 (2.3)		3.03 ± 1.97	2.17 (3.12)		2.12 ± 1.82	1.48 (2.48)		2.83 ± 2.1	2.44 (2.11)	0.006 ****
**Birthweight (gr)**		3487 ± 391	3465 (481)		3547 ± 476	3450 (680)		3587 ± 435	3620 (609)		3755 ± 523	3755 (849)	0.01 *****
**Post-partum bleeding (mL)**		512 ± 363	400 (250)		714 ± 518	500 (425)		552 ± 391	400 (300)		540 ± 465	375 (350)	0.011 ******
**Oxytocine active phase**	16/160 (10.0)			20/52 (38.5)			3/67 (4.5)			13/27 (48.1)			<0.001 #
**Oxytocine secondstage**	61/160 (38.1)			36/52 (69.2)			19/67 (28.4)			21/26 (80.8)			<0.001 ##
**Post-partum oxytocine**	34/160 (21.2)			12/52 (23.1)			20/67 (29.9)			11/27 (40.7)			0.128
**Instrumental delivery**	13/160 (8.1)			10/52 (19.2)			9/67 (13.4)			4/27 (14.8)			0.154
**Post-partum medicine**	15/160 (9.4)			8/52 (15.4)			9/67 (13.4)			5/27 (18.5)			0.423
**Transfusion**	9/160 (5.6)			9/52 (17.3)			6/67 (9)			1/27 (3.7)			0.047 ###

Post-partum medicine (metylergometrine, additional oxytocine, prostaglandines and/or tranexamic acid). * *p* < 0.001 between nullipara with and without epidural; *p* = 0.002 between nullipara without and primipara with epidural; *p* = 0.004 between nullipara and primipara without epidural; ** *p* < 0.001 between nullipara with and without epidural and between nullipara without and primipara with epidural; *p* = 0.002 between primipara with and without epidural; *p* = 0.008 between nullipara with and primipara without epidural; *** *p* < 0.001 between nullipara with and without epidural and between nullipara without and primipara with epidural; *p* = 0.001 between primipara with and without epidural; *p* = 0.019 between nullipara with and primipara without epidural; **** *p* = 0.032 between nullipara with and without epidural; *p* = 0.011 between nullipara with and primipara without epidural; ***** *p* = 0.007 between nullipara without and primipara with epidural (Bonferroni post hoc test); ****** *p* = 0.007 between nullipara with and without epidural; # *p* < 0.001 between nullipara with and without epidural, between primipara with and without epidural, between nullipara without and primipara with epidural, and between nullipara with and primipara without epidural; ## *p* < 0.001 between nullipara with and without epidural, between primipara with and without epidural, between nullipara without and primipara with epidural, and between nullipara with and primipara without epidural; ### *p* = 0.047 between nullipara with and without epidural.

## Data Availability

The data used and analyzed during the current study are available from the corresponding author upon reasonable request.

## Abbreviations

CS	Cesarean section
LSCS	lower segment cesarean section
TOLAC	trial of labor after cesarean
VBAC	vaginal birth after cesarean
